# Socioeconomic gradient shifts in health-related behaviour among Slovak adolescents between 1998 and 2006

**DOI:** 10.1007/s00038-012-0382-9

**Published:** 2012-06-27

**Authors:** Lukas Pitel, Andrea Madarasova Geckova, Sijmen A. Reijneveld, Jitse P. van Dijk

**Affiliations:** 1 Institute of Experimental Psychology, Slovak Academy of Sciences, Dubravska cesta 9, Bratislava, 813 64 Slovakia; 2 Medical Faculty, Kosice Institute for Society and Health, Graduate School, PJ Safarik University Kosice, Kosice, Slovakia; 3 Medical Faculty, Health Psychology Unit, Public Health Institute, PJ Safarik University Kosice, Kosice, Slovakia; 4 Department of Social Medicine, University Medical Center Groningen, Groningen University, Groningen, The Netherlands

**Keywords:** Socioeconomic differences, Health-related behaviour, Adolescents, Smoking, Alcohol, Physical inactivity

## Abstract

**Objectives:**

We aimed to assess the development of the socioeconomic gradient in health-related behaviour (HRB) among Slovak adolescents between 1998 and 2006.

**Methods:**

Data were collected in 1998 (*n* = 2,616; 14.9 ± 0.6 years) and in 2006 (*n* = 1,081; 14.3 ± 0.6 years). ORs of socioeconomic differences—as measured by parental education—were calculated for each cohort in smoking, alcohol consumption and physical inactivity, and the interactions of socioeconomic position and the time period on these behaviours were calculated.

**Results:**

The higher odds of smoking in the low socioeconomic group compared to the high socioeconomic group decreased among boys (interaction OR 0.54), but became evident among girls (interaction OR 1.96). In alcohol consumption, no socioeconomic differences were found among boys, but the higher odds among girls from high socioeconomic position compared with those from low socioeconomic position disappeared in 2006. In physical inactivity, socioeconomic differences increased among boys but not among girls.

**Conclusion:**

During this period, socioeconomic differences in HRB developed in a different way among boys than among girls. Prevalence rates in substance use increased especially among girls from the low socioeconomic group. This group should be particularly targeted by prevention programs.

## Introduction

Socioeconomic (SE) differences in adulthood in health-related behaviour (HRB) have been documented in several studies (Cavelaars et al. 1997; Tyroler 1999; Droomers et al. 1999; Wardle et al. 2003). Usually, the “traditional gradient” is found, i.e. persons with a lower socioeconomic position (SEP) behave less healthily than those with a higher SEP. However, adolescence constitutes a special stage of life during which these differences are absent, smaller or less consistent than in other stages of life (West 1997; Hanson and Chen 2007). In their review of studies on HRB among Western adolescents, Hanson and Chen (2007) found that smoking and especially insufficient physical activity and unhealthy nutritional behaviour were mostly associated with lower SEP, but alcohol consumption and marijuana consumption mostly yielded no associations with SEP. Moreover, socioeconomic differences in HRB may also change over time. Several studies have documented such shifts among the adult population (Luoto et al. 1998; Graham 1996). This has been attributed to such factors as macroeconomic development of the particular societies, aggressive marketing by tobacco corporations (Puska 1997), different stages of the smoking epidemic (Lopez et al. 1994), changes in the prices of the substances (Helakorpi et al. 2010) and others.

However, there is a lack of information about trends in adolescent SE differences in HRB, with the exception of smoking. Since 2000, the Health Behaviour in School-Aged Children (HBSC) reports (Currie et al. 2000, 2004, 2008) have been informing briefly about differences in a range of health behaviours according to family affluence, but the information contained did not allow for a trend comparison.

In time trends regarding HRB among adolescents, gender also seems to play an important role. Pitel et al. (2010) found that between 1998 and 2006, smoking prevalence rates decreased among boys but increased among girls. Kuntsche et al. (2011) found that during the same time period, in most former Communist European countries mean frequency of drunkenness increased among both genders, but much more among girls than among boys. Moreover, there is some evidence from Western countries that gender also may play a role in time trends regarding SE differences in HRB among adolescents. However, the findings are inconclusive. Richter and Leppin (2007) reported a weak positive relationship between parental affluence and smoking among West German boys in 1994 which could not be replicated in 1998 and in 2002, while prevalence rates increased in all SE groups. Among girls, this positive relationship was found only in the 1998 cohort. According to Doku et al. (2010), a positive relationship between parental education and smoking among Finnish adolescents persisted between 1977 and 2007 in both genders. Rasmussen et al. (2009) reported an overall decrease in smoking in Denmark from 1991 to 2006. SE differences fluctuated over time and were different for boys and girls. In Australia, an overall decrease in smoking occurred between 1996 and 2005, and its magnitude did not differ across socioeconomic groups (White et al. 2008).

To our knowledge, no studies from Central Europe exist that explore the relationship between SEP and adolescent HRB over time. The countries of Central Europe underwent a rapid social and economic transition in the 1990s which could have affected the SE gap in HRB, since lower SE groups may have reacted more sensitively to sudden social transitions, as is usual in the case of health indicators (Kunst 1997). The aim of this study is to fill this gap by exploring the development of the SE gradient in smoking, alcohol consumption and physical inactivity among Slovak adolescents between 1998 and 2006. As previous research has indicated that these patterns might be different for boys and girls, each gender will be analyzed separately.

## Methods

### Sample and procedure

Two cross-sectional surveys on adolescents were performed in Kosice (235,000 inhabitants, Eastern part of Slovakia) in 1998 and in 2006, using a similar methodology. In 1998, data were collected among the first year students at secondary schools. The sample was stratified according to the type of school: the proportion of the five educational tracks of the regular Slovak school system was maintained. Individual schools were selected at random. Approximately 20 % of the schools did not wish to participate in the data collection. In the 31 schools which did agree to take part, data were collected in all available classes. A total of 2,616 questionnaires were returned (age range 13.75–17.50 years; mean 14.86 years; SD 0.62; 52.4 % boys).

In 2006, due to changes in the educational system (the introduction of a 9-year elementary education instead of an 8-year one), eighth and ninth year students were approached at randomly chosen elementary schools. In all, 1,081 questionnaires were returned in which gender was specified (age range 13.09–16.83 years; mean 14.33 years; SD 0.62; 47.0 % boys).

In both cases, respondents completed the questionnaire at school, in their classrooms and under the guidance of field workers. The response rates of the students of the schools that participated were 96.3 % in 1998 and 93.0 % in 2006. Non-response was due to illness and other types of absence. Participation in the studies was voluntary and anonymous.

### Measures

*Sociodemographic measures* included gender, age and the highest educational level of parents, which was dichotomized into two levels of education: (1) low SEP, elementary school and apprenticeship or secondary completed with school leaving examinations (boys: 74.5 % in 1998 and 53.3 % in 2006; girls: 77.3 % in 1998 and 61.0 % in 2006); (2) high SEP, university education (boys: 25.5 % in 1998 and 46.7 % in 2006; girls: 22.7 % in 1998 and 39.0 % in 2006).

*Health-related behaviour* included the use of alcohol, smoking and physical inactivity. Respondents were asked how many times they had drunk alcohol in the past 4 weeks (none; 1–2 times; 3–5 times; 6–10 times; 10 times and more), if they had ever smoked a cigarette (no; never; already tried; I smoke from time to time but not daily; I smoke daily now) and how many days per week they performed physical activity. Those who had drunk alcohol three times or more in the past 4 weeks, smoked daily or occasionally and performed physical activity <2 days/week were considered to be engaged in risk behaviour.

### Statistical analysis

Prevalence rates according to gender, year and SEP and high SEP/low SEP odds ratios for the behaviours were calculated. The main effect of SEP, the time period of the study and the interaction of SEP and the time period on HRB (each type and each gender separately) were analysed using a logistic regression model adjusted for age. Data were analysed with SPSS 16.0.

## Results

Among boys, the overall prevalence rates of smoking decreased (Table 1). The prevalence rates increased marginally among boys from the high SE group, but they decreased considerably among their counterparts from the low SE group. While in 1998 boys from the low SE group had statistically significant higher odds compared to those from the high SE group (OR 1.66), this difference had disappeared in 2006. This shift in SE gradient was statistically significant (SEP × period: OR = 0.54). Among girls, the overall smoking prevalence increased (Table 2). This was mainly due to a sharp increase in the low SE group (18.5 % in 1998 vs. 29.6 % in 2006), while also in the high SE group a certain increase was observed. In 2006, the odds of girls from the low SE group were significantly higher when compared with the high SE group. This shift in SE ratio was statistically significant.

**Table 1:** Changes in high socioeconomic position/low socioeconomic position ratio in health-related behaviour between 1998 and 2006 (boys)

	Prevalence in 1998		OR (95 % CI) in 1998	Prevalence in 2006		OR (95 % CI) in 2006	SEP × cohort *z*
	*n*	%		*n*	%		OR (95 % CI)
Smoking (daily or occasionally)							
High SEP	71/346	20.5	1 (ref)	50/232	21.6	1 (ref)	
Low SEP	314/1004	31.3	1.66 (1.21–2.27)**	56/262	21.4	0.88 (0.57–1.37)	0.54 (0.31–0.92)*
Total	385/1350	28.5		106/494	21.5		
Alcohol consumption (3× and more during last 4 weeks)							
High SEP	52/346	15.0	1 (ref)	35/228	15.4	1 (ref)	
Low SEP	141/1006	14.0	0.97 (0.66–1.41)	50/263	19.0	1.25 (0.77–2.04)	1.29 (0.70–2.38)
Total	193/1352	14.3		85/491	17.3		
Physical inactivity (<2× during an ordinary week)							
High SEP	91/346	26.3	1 (ref)	27/226	11.9	1 (ref)	
Low SEP	331/1008	32.8	1.31 (0.99–1.74)	72/259	27.8	2.89 (1.74–4.71)***	2.27 (1.28–4.00)**
Total	422/1354	31.2		99/485	20.4		

*OR* odds ratios adjusted for age, *ref* reference category, *z* this OR indicates the change in time from 1998 to 2006 for low SEP compared to high SEP

* *P* < 0.05

** *P* < 0.01

*** *P* < 0.001

**Table 2:** Changes in high socioeconomic position/low socioeconomic position ratio in health-related behaviour between 1998 and 2006 (girls)

	Prevalence in 1998		OR (95 % CI) in 1998	Prevalence in 2006		OR (95 % CI) in 2006	SEP × cohort *z*
	*n*	%		*n*	%		OR (95 % CI)
Smoking							
High SEP	49/280	17.5	1 (ref)	45/207	21.7	1 (ref)	
Low SEP	176/953	18.5	0.87 (0.60–1.26)	99/334	29.6	1.76 (1.16–2.67)**	1.96 (1.12–3.41)*
Total	225/1233	18.2		144/541	26.6		
Alcohol consumption							
High SEP	38/280	13.6	1 (ref)	27/215	12.6	1 (ref)	
Low SEP	70/949	7.4	0.44 (0.28–0.68)***	44/333	13.2	1.03 (0.61–1.73)	2.40 (1.21–4.75)*
Total	108/1229	8.8		71/548	13.0		
Physical inactivity							
High SEP	160/280	57.1	1 (ref)	62/214	29.0	1 (ref)	
Low SEP	602/952	63.2	1.35 (1.02–1.78)*	111/332	33.4	1.18 (0.81–1.72)	0.87 (0.55–1.40)
Total	762/1232	61.9		173/546	31.7		

*OR* odds ratios adjusted for age, *ref* reference category, *z* this OR indicates the change in time from 1998 to 2006 for low SEP compared to high SEP

* *P* < 0.05

** *P* < 0.01

*** *P* < 0.001

Regarding alcohol consumption, the overall prevalence rate among boys increased only slightly (Table 1). The prevalence rate remained almost the same for boys from the high SE group, but in the low SE group it increased from 14.0 to 19.0 %. However, neither SE differences in either cohort nor the shift in this ratio reached the level of statistical significance. Among girls, the overall prevalence rate in alcohol consumption increased again due to an increase in the prevalence rates in the low SE group (Table 2). Girls from the low SE group drank significantly less often than their high SEP counterparts in 1998, but in 2006 this difference was no longer present. This shift in the SE ratio was statistically significant.

Physical inactivity prevalence rates decreased among boys from both SE groups, but mainly in the high SE group (Table 1). We did not find any low versus high SE group difference in physical inactivity in 1998, but it did appear in 2006. This shift in the male SE ratio was statistically significant. Among girls, a sharp decrease in physical inactivity prevalence rates occurred in both SE groups: from about 60 to 30 % (Table 2). Girls from the low SE group had slightly higher odds to be physically inactive compared to the high SE group in 1998. In 2006, this SE difference disappeared, though this shift was not statistically significant.

## Discussion

We aimed to assess the development of the socioeconomic gradient in smoking, alcohol consumption and physical inactivity among Slovak adolescents between 1998 and 2006. The results of this study reveal that SE differences in HRB among Slovak adolescents changed over time, and different patterns could be found for the different behaviours. Furthermore, different trends in SE differences were found among boys and girls for each behaviour. In smoking, the SE differences decreased among boys but increased among girls; in alcohol consumption, their absence persisted among boys while among girls SE differences disappeared; in physical inactivity, the SE differences increased among boys, but the small differences among girls disappeared, albeit this time trend was not statistically significant.

One possible explanation could be provided by the epidemical approach on substance use (Lopez et al. 1994; Pampel 2001) and by the diffusion-of-innovation theory (Graham 1996): When any trend, positive or negative, is introduced to a society or to any of its segments, such as youth, it is first picked up by males from the elites and only later spreads among females and among people with lower social position. This may explain why the substance use prevalence rates, both alcohol and smoking, barely changed among high SEP boys and girls, but that they changed considerably among low SEP boys and girls. Possibly, the high SEP boys and girls had already undergone the shifts before 1998, and the wave of increase and subsequent decrease of smoking prevalence rates reached the low SEP boys and girls, respectively. Low SEP girls were only then experiencing the phase of increase in 2006, while low SEP boys were already in the phase of subsequent decrease. The general decrease in physical inactivity may be caused by improvements in the system of youth leisure time activities between 1998 and 2006. In the turmoil of the transitional era of the early 1990s, much of the leisure time system collapsed and has had to be re-established since the end of the 1990s.

One argument against the explanation by the stages of smoking epidemic in the population is that, according to Lopez et al. (1994), such a shift takes much longer—about 30 years. Thus, in our study, the epidemic approach could be validly applied only if the substance use epidemic among adolescents were to occur much faster than in the society as a whole. In addition, current information technologies, enhanced electronic communication and electronic mass media may accelerate innovations in behaviours. However, the epidemic approach fails to provide a valid explanation for the changes in physical inactivity, which decreased substantially in both genders and in both groups besides the low SEP boys.

### Strengths and limitations

Besides the excellent response rate, this study provides information about socioeconomic shifts in adolescent health-endangering behaviour from Central Europe, where such studies have been—and still are—very rare. Unlike most previous studies in this field, which only analyzed a single behaviour (usually smoking) (Richter and Leppin 2007; Doku et al. 2010; Rasmussen et al. 2009; White et al. 2008), this study included three distinct behaviours and found a different pattern in each one of them. In addition, we used established measures for all three behaviours, in that way limiting the likelihood of information bias and supporting comparisons with other findings.

A possible limitation is that parental education—the SEP indicator used in our study—may have become less sensitive during the time between the two samples due to a substantial increase of parents with university degree, which was at least to a certain extent spurious, because of official requirements for the employees of Slovak public services. The number of students who graduated from an external study programme (with lectures and classes once a week for students who mostly work full-time during the week) was more than six times higher in 2006 as in 1998 (Institute of Information and Prognoses on Education, Slovakia 2011). Anyhow, the shift is rather large. As we used a rather common indicator of SEP, i.e. parental education, this can easily be compared with future studies. Thus, our findings may even be an underestimation of the changes that occurred. Reijneveld and Gunning-Schepers (1995) provide some hints regarding how to control for demographic shifts which influence the sensitivity of this indicator, if the proportions of the various educational categories in the population change. The latter clearly occurred in Slovakia during this period.

### Implications

In assessing the development of the socioeconomic gradient in smoking, alcohol consumption and physical inactivity among Slovak adolescents between 1998 and 2006, we found that in smoking the SE differences decreased among boys but increased among girls. In alcohol consumption, their absence persisted among boys, but among girls SE differences disappeared. In physical inactivity, the SE differences increased among boys and the small SE differences among girls disappeared, but did not shift significantly. The findings of our study need further confirmation. This could concern both repetitions later over a longer period and replication in other CEE countries, such as Poland, Czech Republic or Hungary. It would also be interesting to compare trends regarding several SEP indicators—parental education, income, affluence as well as status of the adolescents themselves—as they often differ regarding their sensitivity to change (Richter and Leppin 2007). Quantitative and qualitative methods may further be used to gain insight in the processes that have led to these rather big changes.
